# Understanding the complexity of population health interventions: assessing intervention system theory (ISyT)

**DOI:** 10.1186/s12961-021-00743-9

**Published:** 2021-06-19

**Authors:** Linda Cambon, François Alla

**Affiliations:** 1 Chaire de Prévention ISPED/SPF, Université de Bordeaux, Bordeaux, France; 2 Centre Inserm Université de Bordeaux U1219, BPH, Bordeaux, France; 3 CHU Bordeaux, Bordeaux, France

**Keywords:** System, Evaluation, Theory, Public health

## Abstract

Given their inherent complexity, we need a better understanding of what is happening inside the “black box” of population health interventions. The theory-driven intervention/evaluation paradigm is one approach to addressing this question. However, barriers related to semantic or practical issues stand in the way of its complete integration into evaluation designs. In this paper, we attempt to clarify how various theories, models and frameworks can contribute to developing a context-dependent theory, helping us to understand the black box of population health interventions and to acknowledge their complexity. To achieve this goal, we clarify what could be referred to as “theory” in the theory-driven evaluation of the interventional system, distinguishing it from other models, frameworks and classical theories. In order to evaluate the interventional system with a theory-driven paradigm, we put forward the concept of interventional system theory (ISyT), which combines a causal theory and an action model. We suggest that an ISyT could guide evaluation processes, whatever evaluation design is applied, and illustrate this alternative method through different examples of studies. We believe that such a clarification can help to promote the use of theories in complex intervention evaluations, and to identify ways of considering the transferability and scalability of interventions.

## Introduction

Experimental designs are popular in health research because of their high internal validity [1]. This internal validity is related to the ability to control confounding variables (i.e., high internal validity gives substantial confidence that the results are due to the intervention itself). To limit such confounding variables, the experiment must employ principles of “all things being equal” (e.g., population characteristics and external factors) and high intervention standardization, which are in fact remote from real-life conditions (e.g., delivery modalities, stakeholder compliance and patient selection). This results in universal laws that are free from contextual influences, considered as bias [2].

However, population health interventions (PHIs) are generally considered complex, as they include several components which interact with one another to produce a number of outcomes [3]. Moreover, beyond the interventional components, the intervention should not be isolated from the specific context in which it is implemented [3–5]. Indeed, rather than an intervention, it should be considered an “interventional system” [5, 6] that includes pre-existing contextual parameters that could be within or outside the control of intervention developers and implementers. Hence, an evaluation should assume that (i) the contribution of all components in this interventional system, as well as the effect of their combination, must be evaluated, and (ii) the conclusions of the study/trial are context-based, (iii) even though some of the conclusions (i.e., the key functions) could be transferable to other settings [12].

The question thus becomes: How should the effect of individual components of this interventional system and their interactions be evaluated to identify the key functions? Such an analysis is necessary to define the conditions of intervention transferability and scalability.

One way to do this would be to theorize interventions by using the theory-driven evaluation (TDE) paradigm [6, 7]. Indeed, a TDE [8–10] is based on a contribution analysis [11] which assesses questions inferring causality in real-life programme evaluations [12]. The aim is to reduce uncertainty about the contribution of all input that could contribute to the outcome. A TDE could be an evaluation design on its own as an alternative to an experimental trial (e.g., a realist evaluation), or it could be combined with/integrated into a classical experimental design [7, 13, 14]. The core principle is to base the evaluation on an explicit conceptualization of the theory used to define the data collection, such that the theory conceptualizes the features of the intervention that should be made explicit and validated by the evaluation process.

What theory are we talking about in TDE, however? Indeed, while various methodological studies have acknowledged that this theory-based approach is crucial [7, 16], they have also noted the tendency to pick a theory “off the shelf” rather than use task-specific theories of change, even as many dominant theories “have done little to make interventions more effective” [16]. These points remind us of the need to pay careful attention to evidence-based arguments when selecting a theory [16]. Similar to other authors [7], they specify that the issue of integrating TDE more fully into an evaluation design involves clarifying what we actually mean by theory as “people are talking at cross purposes in relation to the various kinds of theory” [7]. We hypothesize that if TDE is underused in PHI research (PHIR) [15], it is due to the failure to define the so-called theory and the lack of clear and practical guidelines for designing and validating this theory.

This article therefore aims to further the use of TDE through two pragmatic suggestions based on our practical experience, namely, clarification of (i) what the “theory” in TDE could be and (ii) how it might be employed in intervention development and evaluation designs.

### Designing and qualifying a theory in TDE

A theory has been defined as “a set of analytical principles or statements designed to structure our observation, understanding, and explanation of the world” [16]. This definition is broad and can generate confusion. Attempting to clarify the definition in the field of science implementation, Nilsen [16] proposed three main conceptualizations: a theory can be described as explanatory, “made up of definitions of variables (…) and a set of relationships between the variables and specific predictions”; a model can be described as descriptive, not explanatory, and as providing a “deliberate simplification of a phenomenon or a specific aspect of a phenomenon”; or it can be seen as a framework, which is also descriptive, but not explanatory, “presenting categories (e.g., concepts, constructs, or variables) and the relations between them that are presumed to account for a phenomenon”. Nilsen describes five theoretical approaches in implementation science: process models, determinant frameworks, classical theories, implementation theories, and evaluation frameworks [16]. The differences between these definitions remain slight, and some crossover exists between them. For example, some of the “classical theories” (i.e., essentially psychosocial theories) are called “models”, despite their offering explanations, such as the health belief model [17]. Evaluation frameworks (e.g., TDE) provide another example, describing the steps involved in conducting an evaluation. These can also be defined as process models dedicated to evaluation .

In this context, it is not easy to clarify a theory in TDE; is it one of the abovementioned theories, models or frameworks? Or a combination of them? In TDE, the theory explains how a programme generates its effects (why and how the intervention works) by defining a set of explicit or implicit assumptions on the part of stakeholders about which action is required to solve a problem and why the problem will respond to this action [9, 18, 19]. Following on from our previous work on interventional systems assuming this blurring between context and intervention components [5, 20], the theory in TDE should integrate various elements arising from other theories, models and frameworks because (i) it is explanatory, considering which causal pathway is expected to meet the goal, similar to classical theories; (ii) it hypothesizes which specific actions and sequences of implementation contribute to this causal pathway, similar to a process model; and (iii) it considers contextual elements and their influence within a specific setting. To be clear, we took on board Moore and Evans’s statement [21]: “[A] single theory will not tell the whole story because it could place weight on some aspects (e.g., certain causal factors) at the expense of others.” From this perspective, excluding one theoretical approach in favour of another could lead to a partial understanding of the intervention system; the theory in TDE should consider all of these theoretical approaches as long as they are well understood and differentiated. Therefore, we suggest introducing the concept of interventional system theory (ISyT), which would combine what we define as:

- Causal theory (the term “theory” was chosen because of its explanatory aspect and its independence from a classical theory): Causal theory involves explanatory and mechanistic components, but it also considers all of the determinants likely to be involved as barriers or enablers to meet goals, as well as actions triggering the expected mechanisms.
- Action model (the term model was chosen because of its sequential pattern, as in a process model): Action models are employed by developers and implementers. They provide concrete elements of action and implementation intended to guide the process to meet the purposes. The core aspect of the action model is its focus not only on the activities involved in outcomes, but also on the sequences, resources and prerequisites needed to implement them.

**Fig. 1:**
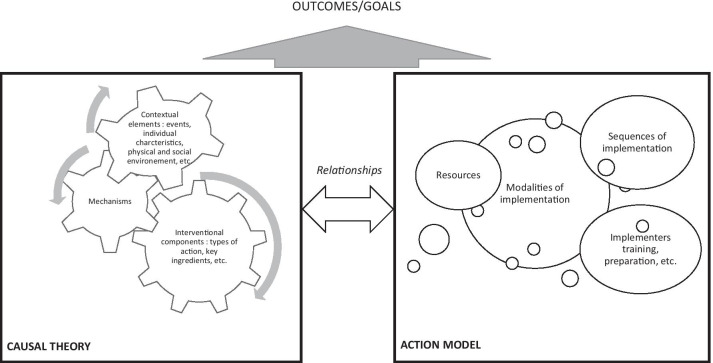
Interventional system theory

Figure 1 describes ISyT.

### Articulation of various theoretical approaches with ISyT

Once the ISyT has been defined, the question of its articulation with existing theories, models and frameworks arises. In fact, we suggest that all of its components could be informed by these theories a priori. Indeed, a causal theory could be informed by explanatory theories such as classical theories (e.g., mechanisms and causal relationships between variables) or by determinant frameworks (contextual influencing parameters), and the action model by implementation theories or process models.

For example, one of the mechanisms involved in behavioural change is motivation (a mechanism), which is enhanced by self-efficacy (another mechanism). Motivation and self-efficacy and their influence on behaviour (goal) have been documented in numerous classical theories. One way to increase self-efficacy is to provide positive feedback on the change process (interventional component), an approach that has been documented by several implementation theories. This positive feedback could be provided by professionals (another interventional component), but also by relatives or communities around the person, who need to be involved and sensitized to support the person in the change process (another interventional component). Some experiments have explained how to mobilize these communities according to specific or generalizable process models that involve training or supportive processes. The ability to do this may be dependent on multiple contextual elements that can act as barriers or enablers (contextual elements). For instance, the motivation to change could be impeded or favoured by the opportunity for change due to a lack of, or the provision of, resources to support the change. The roles of these contextual factors have been documented by numerous socioecological determination frameworks [22–25].

Hence, we attempt to synthesize these different approaches (framework models, classical theories, etc.) in the field of PHI to assess how they might help to clarify theoretical understanding of the intervention system. This work is summarized in Table 1, which clarifies the contributions of these theories to an understanding of the interventional system, thereby providing a definition of the components of the ISyT. We did not include the methodological frameworks that are potentially useful to define the development and evaluation stages of ISyT (such as TDE frameworks).

**Table 1:** Four theoretical approaches for understanding the population health interventional system (ISy)

Terms	Definition	Constructs	Purpose	Specificities	Examples in the public health field	Value in understanding intervention systems
Determinant framework	An overview of determinants and categories presumed to account for a phenomenon by acting as barriers and enablers	Environmental determinants Sociological determinants Psychological determinants Organizational determinants	Providing clues as to how the micro–meso–macro context could influence a health phenomenon	Multilevel With multiple influences Provides no explanation, only clues Derived from empirical studies of barriers and enablers	Social determinant frameworks [22]	Identifying all of the elements to be considered in understanding the system from multilevel points of view
Classical theory	An explanatory definition of relationships between variables and the specific results of their combinations	Psychosocial constructs Structural constructs Relationships among all constructs and specific predictions, especially those formulated as mechanisms	Explaining how and why specific relationships among a set of constructs lead to specific events	Focused on the mechanisms of effects Provides some explanations Derived from fundamental work in various disciplines (psychology, sociology, political sciences, etc.)	Behavioural: social cognitive theory [26] Organizational/social: social capital theories [27]	Identifying the mechanisms of effects and the factors potentially involved in their triggering
Process model	A deliberate simplification of a process describing how different resources could be combined to produce a change within a specific context	Variables relating to implementation (training, communication, decision, revision, etc.) May include some contextual elements influencing the delivery	Describing and/or guiding a process	Recognizing a temporal sequence and conditions of the progression of implementation endeavours More or less emphasis on the context and its influence on delivery Derived from field expertise and experimentation	The PRECEDE–PROCEED model [28]	Identifying the combination of resources and activities, as well as their sequence, needed to produce a change
Implementation theories	A combination of classical theories and activities, with or without a temporal sequence	Implementation Constructs involved in mechanisms triggering effects Mechanisms of effects	Explaining how and why specific relationships between a set of constructs and interventional elements lead to specific events	Derived from field expertise and experimentation Derived from fundamental work in various disciplines (psychology, sociology, political sciences, etc.)	The behaviour change wheel [29]	Linking mechanistic hypotheses and the resources and activities potentially influencing them to design or understand how interventional inputs could work

### The role of mechanisms in ISyT

The aim in designing this ISyT is to understand how these mechanisms are produced [5, 6] and under which conditions they trigger specific results. Such mechanisms are consequently considered key functions of the interventional system [5, 6]. Their integrity guarantees the transferability of an intervention. We should distinguish these key functions from their form which reflects adaptation to the context, at the same time noting that there are different definitions of mechanisms [5, 26]. Machamer et al. [27] defined them as “entities and activities organized such that they are productive of regular changes from start or set-up to finish or termination of conditions”. Weiss defined them as different from activities, but the response that the activities generate [8, 28]. In a similar vein, in the realistic approach, a mechanism is “an element of reasoning and reaction of an agent with regard to an intervention productive of an outcome in a given context” [26]. In health psychology, they can be defined as “the processes by which a behavior change technique regulates behavior” [29]. This could include, for instance, how practitioners perceive an intervention’s usefulness or how individuals perceive their ability to change their behaviour.

These definitions have a common point: mechanisms are the inescapable prerequisites for change. In this respect, we argue that they are key factors for investigation by means of a TDE, and are the elements of an interventional system that must be reproduced during transfer to other settings [5, 6]. Indeed, many combinations of intervention–contextual elements could produce the same mechanism (e.g., some people are sensitive to emotional support during smoking cessation, while others prefer to have technical support, but both types of support can trigger the same mechanism: i.e., the impression of being reassured, helped and supported). During the transfer to other settings, implementation variations, population characteristics and organizational factors can change, producing the same mechanism or mechanisms that differ from those expected. The intervention process could be adapted to each new context if these adaptations permit the expected mechanisms to occur, which is why the mechanisms are the key functions that must be reproduced.

Thus, the characteristics described in Table 2 can be attributed to ISyT.

**Table 2:** Characteristics of ISyT

Characteristics	
An explanatory purpose	Hypothesizing how intervention works within a context
A pragmatic role	Guiding how one should act to achieve a goal
A broad understanding of each element likely to influence outcomes	Including a systemic approach intervention/context
Context based	Conceived as a grounded theory describing all parameters in play in a specific context
Potentially generalizable	Highlighting the mechanisms of effect, which are conceived as the key functions of the intervention

### Using ISyT in the evaluation process

**Fig. 2:**
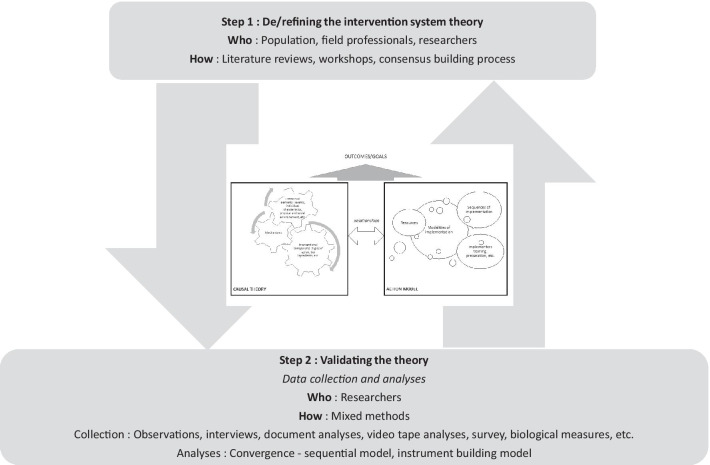
Using ISyT in the evaluation process

With reference to the theory-driven paradigm, ISyT contributes to the design and evaluation of an interventional system. As in the various theory-driven frameworks such as realistic evaluation [30] or the theory of change (TOC) framework [10], the process involves various key steps in articulating definitions, revising theory and collecting data to inform and refine the theory. To be complete and effective, this process should (i) be established in a participatory way that combines experiential and scientific knowledge, and involves different stakeholders, including populations targeted by the intervention, field professionals developing the intervention who are aware of the context, and researchers providing a global and multidisciplinary analysis of the phenomenon under study; (ii) employ an evidence-based and rigorous consensus-building process that includes, for example, literature reviews, workshops and exploratory studies; (iii) use a hybrid approach that combines both hypothetico-deductive and inductive methods; and (iv) mobilizes quantitative and qualitative data collected in mixed-methods designs [31, 32]. This process could be defined as that depicted in Fig. 2.

The way in which ISyT is developed depends on the subject, the actors involved (field professionals and population), the extent of their participation in the research process, and the point at which this development takes place in the intervention development process (e.g., to be developed, partly developed, already implemented, or an adaptation of an existing intervention). It is thus difficult to specify which method to use, except that, ideally, it should combine data from several sources: (i) data to explain the mechanisms to be activated (fundamental research, actors’ expertise, pre-existing theoretical frameworks), (ii) data to explain the influence of environments, actors and organizations on these mechanisms (determinants of change, existing and effective means of influencing these determinants), and (iii) data on the feasibility and acceptability of intervention components enabling the intervention inputs to be adjusted (perception of actors, concrete elements concerning the mobilization of resources to be used in order to promote the applicability of intervention components over time). These data must make it possible to understand all the components of the system to be considered and, as far as possible, to anticipate the way in which they interact with each other. This involves both combining reviews of the scientific literature with an analysis of reports or results on pre-existing interventional levers or programmes, qualitative investigations (interviews or observations) with the actors involved, and consensus-building processes in order to stabilize the hypotheses put forward by ISyT.

In the *Transfert de Connaissances en REGion* (TC-REG) study [40], the theory elaboration process involved two major steps. In step 1, we defined the initial middle-range theory and the knowledge-transfer scheme through (i) a literature review of evidence-based strategies for knowledge transfer and mechanisms to enhance evidence-based decision-making (e.g., the perceived usefulness of scientific evidence); (ii) a qualitative exploratory study in the four regions under study to collect information on existing actions and resources to implement knowledge-transfer strategies; and (iii) a workshop with experts in knowledge transfer, field professionals from the four regions, and TC-REG researchers to select the strategies to be implemented and develop hypotheses regarding the mechanisms potentially activated by these strategies together with any contextual factors potentially influencing them (e.g., the availability of scientific data). In step 2, we validated the initial middle-range theory through two qualitative studies, the first to identify specific actions implemented in the regional knowledge-transfer plan (one series of interviews), and the second to identify the final middle-range theories through two series of interviews and a workshop with the same stakeholders in order to elaborate the theory by combining strategies, contextual factors and the mechanisms to be activated.

**Fig. 3:**
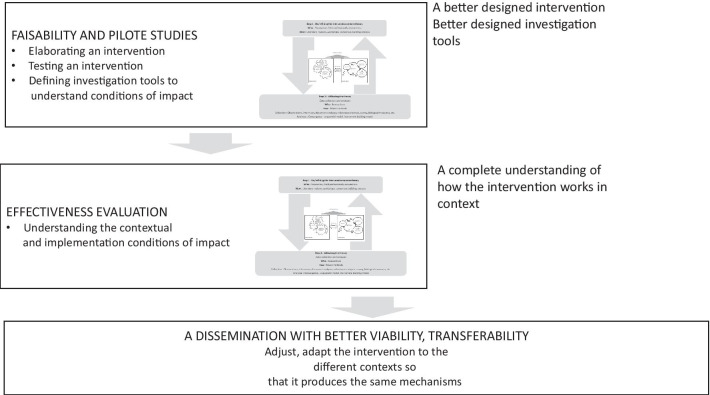
Articulating experimental design and ISyT

This process is not incompatible with the different stages of experimental evaluations, whatever the stages of their development might be. Indeed, the process could be combined with experimental designs [15]. Figure 3 presents the potential articulation between the intervention system and the experimental design based on the three major steps of trials outlined in the Medical Research Council guidelines [3]: pilot study, evaluation of effectiveness and dissemination. Associating a theory-based approach in pilot studies and subsequent effectiveness studies contributes to a better intervention and evaluation design [7], as well as a better understanding of how the intervention works in the designated context. Both contribute to enhanced dissemination of the intervention given its improved viability under real conditions [33].

We used this process in the OCAPREV [*Objets connectés et applications en prévention*] study, for example, to design an evidence- and theory-based intervention, namely a health application, in a pilot study prior to an evaluation [34]. In the ee-TIS [e-intervention Tabac Info Service] study [35], we conducted a randomized control trial with a contribution analysis evaluating a smoking-cessation app (Tabac Info Service). In addition to evaluating outcomes, the study sought to understand how each component of the app encouraged smoking cessation through the mechanisms triggered (e.g., self-efficacy, utility perception, confidence in the app) and the contextual parameters potentially influencing smoking cessation (e.g., smoking status of the domestic partner; presence of children; others’ support in smoking cessation; family, social or professional events).

Moreover, our process is not incompatible with other theory-driven frameworks such as TOC or realistic evaluation frameworks. For example, according to TOC, interventional components or ingredients are fleshed out and examined separately from the context. The initial hypothesis (the theory of change) is based on empirical or theoretical assumptions. Validation (or its absence) then addresses the extent to which the explanatory theory, including the implementation parameters, corresponds to observations. This explains how input, activities and outcomes are linked and how the various interventional components work together in a causal pathway involving causal inferences and implementation aspects to achieve the impact [10]. The difference from our approach is the lack of focus on mechanisms and on the influence of context; instead, in TOC frameworks, the focus is on the intervention and its specific components. In the systemic approach of an ISyT, contextual elements and the mechanisms of effects are actually included in the matrix [5]. This is what we did in the OCAPREV study, where the theory hypothesized 50 causal chains linking behavioural sources (capacity, motivation, opportunity to change) with specific behaviour-change techniques and mechanisms of effect. Some technical recommendations (i.e., implementation processes or contextual elements) were added to these chains to improve the app’s accessibility, acceptability and contribution to reducing health inequalities.

In realistic evaluations [30], contextual elements and mechanisms are considered core elements in middle-range theories. Interventional and implementation components, which are included in the ISyT, are not taken into consideration. Some authors have proposed including these interventional components, called “resources”, in the definition of mechanisms [36]. We do not share this opinion, however, preferring instead the definition of mechanism suggested by Lacouture et al. [26], who focused on the reaction of stakeholders situated in the context (including their interventional input). Thus, according to our definition, what Dalkin [36] termed resources is an aspect of contextual (pre-existing resources) and interventional components (resources provided by creators and implementers), rather than part of the triggering mechanisms. Others have distinguished between intervention and context by referring collectively to the intervention, context, actors, mechanisms, outcomes (ICAMO) configurations [40]. Our interventional system approach does not share this perspective, which blurs the distinction between intervention and context and disregards our position that actors are part of the context. We prefer to keep the tryptic C (context)–M (mechanism)–O (outcome) model by adapting it as follows: Ce (context external to the intervention)–Ci (interventional context)–M (mechanism)–O (outcome). For example, the goal in the TC-REG project [37] was to evaluate the conditions of effectiveness of a knowledge-transfer scheme aimed at evidence-based decision-making (EIDM) in different public health organizations. The middle-range theories were made up of external factors (e.g., initial training of implementers, interest in knowledge-transfer scheme dissemination, leadership profile, political support in the organizations, time to study evidence-based data, team size) (Ce = context external), interventional components (e.g., access to evidence-based data, training courses, seminars, knowledge brokering) (Ci = context interventional) and mechanisms (M) triggered by the combination of both (perception of EIDM utility, incentive to make evidence-based decisions, self-efficacy to analyse and adapt evidence in practice, etc.) to produce outcomes (O).

**Fig. 4:**
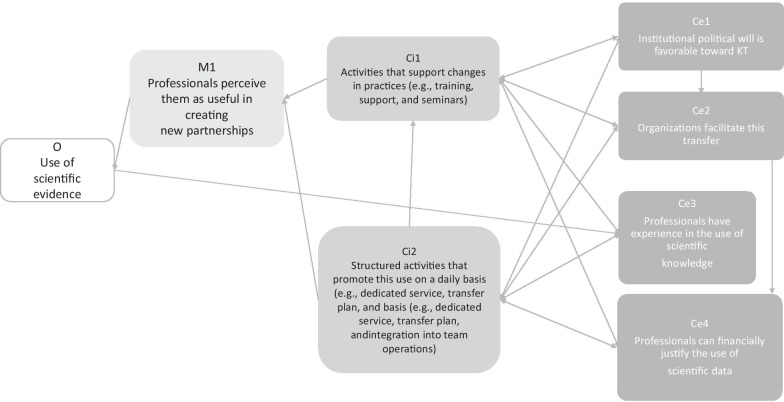
An example of final middle range theory in TC REG Study

This process resulted in the creation of eight final middle-range CeCiMO theories about the mechanisms triggered and combined in a final ISyT, leading to the use of scientific evidence. Eight mechanisms were identified to achieve this goal, for example, “professionals perceive them as useful to legitimize or advocate for their professional activity”. Each was triggered by a combination of knowledge-translation activities (interventional components) and contextual components that influenced the activities and also directly influenced the mechanisms, for example, “political will in favour of knowledge translation”; “professionals are aware of the dissemination channels”; “political will and professionals’ experience”; and “favourable organizational conditions”. Figure 4 shows an example of CeCiMO in the TC-REG study.

### ISyT and other programme theories

The major difference of ISyT compared to other research work is the place of contextual elements which are completely integrated in a circular causal perspective, fully in line with the systemic approach as embodied by the principle of realistic evaluation. This approach is brought into effect by interactions between the different components in the system—contextual and interventional and mechanistic—simultaneously modified by and modifying one another.

Carol Weiss, for example, introduced the notion of theory of change, defined as “the set of assumptions that explain both the mini-steps that lead to the long term goal of interest and the connections between program activities and outcomes that occur at each step of the way” [38]. For her, the theory of change involved two components: an implementation theory (i.e., descriptively forecasting the steps to be taken in implementing the programme) and a programmatic theory (i.e., a theory based on the mechanisms that make things happen) [8]. ISyT could be considered as a theory of change according to Weiss’s definition, with the major difference that our causal theory introduced some contextual components in addition to, and in interaction with, the activities.

Funnell and Rogers [39] also added many clarifications to the different terms used in the TDE. They proposed clarifying: (i) the programme theory, defined as an explicit theory of how an intervention is understood to contribute to its intended or observed outcomes—ideally, it includes a theory of change and a theory of action (NB: different from Weiss’s definition); (ii) the theory of change, defined as the central processes or drivers by which change comes about for individuals, groups or communities, derived from a research-based theory of change or drawn from other sources; (iii) the theory of action, defined as the ways in which programmes or other interventions are constructed to activate these theories of change; and (iv) the logic model, defined as a representation of a programme theory, usually in the form of a diagram [39]. According to these definitions, ISyT should be a programme theory with the inclusion of contextual elements in the theory of change (according to their definition), in addition to the theory of action (input from the intervention and sequenced processes to achieve outcomes) (the action model in ISyT).

In a third example, Chen’s action model/change model schema [29], ISyT distributes its contextual components differently. For Chen [29], the change model is related to the specific area of intervention in a linear causality perspective, associating the intervention activities, the determinants and the outcomes they are supposed to impact. The action model is a systematic plan for arranging staff, resources, settings and support organizations in order to reach a target population and deliver the intervention services. Described as a “programmatic model” (different from Weiss’s definition), it sets out the major aspects a programme needs to secure: ensuring that the programme’s environment is supportive (or at least nonhostile), recruiting and enrolling appropriate target group members to receive the intervention, hiring and training programme staff, structuring modes of service delivery, designing an organization to coordinate efforts, and so on. Once again, the difference lies in the place and role of the system’s contextual components. In ISyT, they should not, as in Chen’s schema, be considered only through the implementation of the intervention (Chen’s action model), but also in the production of the effects (Chen’s change model), whether they are manipulable by the implementers (as suggested by Chen’s model), or not (as evoked in ISyT, such as the intrinsic characteristics of organizations or individuals). Here again, the difference lies in the search for feedback loops (as in realistic evaluation), where Chen’s schema proposes a linear reading of events.

Finally, ISyT attempts to contribute to Hawe and colleagues’ work on systems-thinking in the field of prevention [40]. The authors stressed the need to focus on the dynamic properties of the context in which the intervention is introduced via three dimensions: (i) their constituent activity settings, (ii) the social networks that connect the people and the settings; and (iii) the time. They consider the intervention as a “critical event in the history of a system, leading to the evolution of new structures of interaction and new shared meanings”. ISyT fits neatly into this approach through its attempt to add pragmatic methodological elements to untangle the different dimensions.

## Conclusion

It is essential to develop a deeper understanding of what is happening inside the “black box” [41] of PHIs. One way to do this is through a theory-driven intervention/evaluation paradigm. However, several barriers have delayed its full integration into public health evaluation designs, especially when we consider an interventional system within a context rather than simply as an intervention. In this case, the evaluation concept should fit in with core aspects of the system [42], notably, the interactions between elements (the relationship between two elements is never unilateral), the holistic nature of the system (the system cannot be reduced to the sum of its parts), its organization (the system is an agent of a relationship that produces behaviours different from those produced by each component individually), and the fact that its complexity derives from the system itself as well as from the uncertainties and its inherent vagaries, randomness, and so on. These characteristics mean that we need to define the theory that will be adopted to represent this system. We thus suggest developing an ISyT (i.e., a context-dependent theory, that is different from classical theories, models or frameworks, but is nonetheless informed by them) and conceptualizing how it could be used and articulated in different evaluation designs, such as experimental trials or other TDE frameworks. This type of clarification could help encourage the use of theories in complex intervention evaluations and shed light on ways to consider the transferability and the scalability of interventions.

## Data Availability

Not applicable.

## Abbreviations

TDE: Theory-driven evaluation
ISyT: Intervention system theory
PHIR: Population health intervention research
TC-REG: Transfert de Connaissances en REGion
TOC: Theory of change
CeCiMO: Context external–context interventional–mechanism–outcome
